# Prosody in the Auditory and Visual Domains: A Developmental Perspective

**DOI:** 10.3389/fpsyg.2018.00338

**Published:** 2018-03-19

**Authors:** Núria Esteve-Gibert, Bahia Guellaï

**Affiliations:** ^1^Departament de Llengües i Literatures Modernes i d’Estudis Anglesos, Universitat de Barcelona (UB), Barcelona, Spain; ^2^Laboratoire Ethologie, Cognition, Développement, Université Paris Nanterre, Nanterre, France

**Keywords:** speech, gestures, prosody, development, multimodality

## Abstract

The development of body movements such as hand or head gestures, or facial expressions, seems to go hand-in-hand with the development of speech abilities. We know that very young infants rely on the movements of their caregivers’ mouth to segment the speech stream, that infants’ canonical babbling is temporally related to rhythmic hand movements, that narrative abilities emerge at a similar time in speech and gestures, and that children make use of both modalities to access complex pragmatic intentions. Prosody has emerged as a key linguistic component in this speech-gesture relationship, yet its exact role in the development of multimodal communication is still not well understood. For example, it is not clear what the relative weights of speech prosody and body gestures are in language acquisition, or whether both modalities develop at the same time or whether one modality needs to be in place for the other to emerge. The present paper reviews existing literature on the interactions between speech prosody and body movements from a developmental perspective in order to shed some light on these issues.

## Introduction

Human language is an interesting input as it can be perceived through both ears and eyes. For example, adults’ comprehension of speech in noisy and quiet environments is enhanced when they have access to the visual cues conveyed by the speaker’s face (Sumby and Pollack, 1954). In face-to-face interactions, the whole body is involved and may serve informative purposes (Kelly and Barr, 1999 for a review; Kendon, 2004). People around the world produce spontaneous gestures while talking. These gestures accompanying speech, called ‘co-speech gestures,’ are so connected with speech that people use their hands even when nobody sees them (Corballis, 2002), and congenitally blind people gesture when interacting with each other (Iverson and Goldin-Meadow, 1998 and Goldin-Meadow, 1998). Gestures can be defined on the basis of the articulator that is being used to produce them (the head, as in head nods or head tilts; the hand, as in manual pointing, manual beats or iconic gestures; the face, as in oral gestures or in facial expressions such as eyebrow movements), on the basis of whether or not they are accompanied by speech (co-speech gestures), or based on whether the gesture movement is continuous or discrete (see Wagner et al., 2014 for a review). Another order of things is the function for which they are used in language and communication. Gestures can serve a deictic or highlighting function, they can depict and represent semantic meanings, and they can structure information in the discourse and be an indicator of pragmatic implicatures to be driven for a successful communication to take place. Because all these levels have parallels with the prosodic properties of speech, these gestures are also called visual correlates of prosody.

It is now clear that co-speech gestures fulfill multiple cognitive functions. Some studies focused on speaker-directed functions suggesting that gestures may ease the speaker’s cognitive load (Cook and Goldin-Meadow, 2006; Chu and Kita, 2011), promote learning (Ping and Goldin-Meadow, 2010), help in the conceptual planning of information and discourse (Alibali et al., 2000; Cutica and Bucciarelli, 2008), and facilitate lexical access (Rauscher et al., 1996; Alibali et al., 2000). Others stress that gestures enhance the transfer of information by providing it cross-modally, thereby facilitating uptake for addressees (De Ruiter et al., 2012; Guellaï et al., 2014). These proposals account for the adults’ use of co-speech gestures and focus on gestures with a referential value in communication (deictic and iconic hand movements). Yet, they are less effective for explaining developmental patterns as well as the role of gestures with a non-referential value in communication (such as facial expressions and rhythmic ‘beats’).

In the following sections we propose to explore the developmental links between speech and body movements (i.e., hand and head gestures, and facial expressions), focusing on one specific linguistic aspect, namely prosody. Prosodic properties of speech encode prominence, phrasal organization, speech act types, emotions, attitudes, and beliefs (e.g., Pierrehumbert and Hirschberg, 1990; Ladd, 1996; Byrd and Saltzman, 2003; Jun, 2005). There is a growing body of research showing that prosody is not only expressed through the tonal and temporal properties of speech, but also by means of body movements produced with the hand, head, or face (e.g., Krahmer and Swerts, 2007; Cvejic et al., 2012; Guellaï et al., 2014). The speech and gesture dimensions of prosody are found to be tightly intertwined at the temporal, semantic, and pragmatic levels, and this is true not only in adult speech but also in language development.

Speakers’ body movements are temporally coordinated with the prosodic structure in speech, pitch accents and boundary tones serving as anchoring points for prominent phases in body movements (Hadar et al., 1983; De Ruiter, 1998; Leonard and Cummins, 2011; Esteve-Gibert and Prieto, 2013; Ishi et al., 2014; Ambrazaitis and House, 2017; Esteve-Gibert et al., 2017a). At the semantic and pragmatic levels, prosody and gestures can both have a deictic component through which speakers highlight certain elements in speech (Levelt et al., 1985; Roustan and Dohen, 2010), they can disambiguate syntactic constituents (Guellaï et al., 2014; Krivokapic et al., 2016), and mutually influence the processing of speaker’s emotions, beliefs, and attitudes (Ekman, 1979; Kendon, 2004; Poggi et al., 2013). In the multimodal expression of prosody, the gesture dimension can consist of movements of the hand or head, facial expressions, or body postures. Traditionally, different types of body movements have been studied independently (for instance, facial expressions have received more attention in the literature on emotions, while hand movements have been the focus of studies on the referential value of gestures in language). In the present paper we will refer to these different types of movements as ‘gestures,’ as we propose that it is more interesting to take them as a whole to have a complete picture of the speech-gesture relationship in language and communication development.

## Temporal Aspects of the Audio–Visual Speech Integration in Infancy

Infants need to make sense of the rich multisensory stimulations present in their everyday experiences. From the earliest stages of development, infants are found to relate phonetic information from the lips and the voice (Kuhl and Meltzoff, 1984; Aldridge et al., 1999; Patterson and Werker, 2003). In these studies, infants were presented with videos, side-by-side, of two faces articulating two vowels (i.e., /i/ vs. /a/), while hearing only one vowel (i.e., either /i/ or /a/). Infants are considered to be able to detect audio–visual congruency if they look longer at the matching stimulus. Remarkably, there is evidence that from birth, infants detect equivalent phonetic information in the lips and voice (Aldridge et al., 1999). Auditory-visual phonetic matching is also shown at 2 months (Patterson and Werker, 2003), at 4 months and a half (Patterson and Werker, 1999), and at 8 months based on the gender of the talker (Patterson and Werker, 2002). When the vowels are reduced to sine-wave analogs or simple tones, infants do not detect the congruent video anymore (Kuhl et al., 1991). Taken together, these studies, focusing on perioral and facial cues, suggest that infants already have the primitives of lip reading for single speech sounds.

On the production side, newborns bring their hands and objects to their mouth, and explore them orally, these behaviors being considered to be the earliest signs of the oral-manual link in language development (Iverson and Thelen, 1999). Around 6–7 months of age infants start to babble, a rhythmic close–open movement of the jaw that results in the production of syllables (Oller, 2000; Vihman et al., 2009). At the same age infants start producing rhythmic arm movements that are temporally aligned with the vocal babbling (Ejiri, 1998; Iverson and Fagan, 2004). Interestingly, the acoustic quality of the infants’ babbles improves when infants combine these vocalizations with rhythmic arm movements, as syllables become shorter and display shorter formant-frequency transitions (Ejiri and Masataka, 2001).

The time-aligned coordination of gesture and speech is also present at later stages of language development. At the onset of word production infants start combining vocalizations with pointing gestures signaling referents in space, and these gestural and speech dimensions are timely aligned in an adult-like way: the accented syllable in speech coincides with the apex of the pointing gesture (Butcher and Goldin-Meadow, 2000; Esteve-Gibert and Prieto, 2014). Later, at 4–5 years of age we observe the emergence of bi-phasic body movements that have no referential meaning and that are timed with pitch accents that children use to emphasize specific information in the sentence (Nicoladis et al., 1999; Capone and McGregor, 2004; Esteve-Gibert et al., 2017b; Mathew et al., 2017). These movements are typically produced with the hand, arm, or head, and are called beats in the gesture literature (Kendon, 2004; McNeill, 2005; Wagner et al., 2014). Beats provide clear evidence of the rhythmic entrainment between the acoustic and visual dimensions of language, because speakers are found to necessarily modify the acoustic properties of speech when they produce these body movements (Krahmer and Swerts, 2007). Thus, prosodic structure seems to be observed at the speech and at the gestural levels, both dimensions being temporally aligned in a precise way from early stages of language development.

## Implications of the Audio–Visual Integration for Word Learning

When addressing infants, adults usually use a speech register which is commonly called Infant-Directed Speech (IDS). This speech register has been the focus of numerous studies as it presents particularities in the auditory domain. It is characterized by slower speech rate and exaggerated pitch excursions compared to Adult-Directed Speech (ADS) (e.g., Fernald and Simon, 1984; Grieser and Kuhl, 1988; Fisher and Tokura, 1995). Vowel and consonant contrasts are more clearly produced in IDS, and this acoustic difference helps infants to build their phoneme inventories (Kuhl et al., 1991; Werker et al., 2007; Cristia, 2011). Also, the slower speaking rate and vowel properties help 21-month-olds learn and remember new words better (Song et al., 2010; Ma et al., 2011).

It has also been observed that IDS is associated with exaggerated facial cues: when addressing infants, caregivers usually exaggerate facial expressions and articulatory lip gestures for corner vowels (Chong et al., 2003; Green et al., 2010). It has been argued that visual IDS attracts infants’ attention to the speaker and helps them to parse the speech stream (Kitamura and Burnham, 2003). Some authors have examined sensitivity to the temporal synchrony of visual prosody using continuous IDS (Blossom and Morgan, 2006). They found that infants aged 10–11 months use visual prosody to extract information about the structure of language as they matched synchronous faces and voices. More recently, it has been shown that 8-month-old infants reliably detect congruence between matching auditory and visual displays of a talking face based on prosodic motion (Kitamura et al., 2014), and that 9-month-olds can detect whether a manual deictic gesture is congruently aligned with the corresponding speech segment (Esteve-Gibert et al., 2015). Using an intermodal matching paradigm, Kitamura et al. (2014) presented 8-months-old infants with two visual displays of talking faces (i.e., only moving dots) and one utterance that matched one of the two facial configurations. Results showed that infants reliably detect auditory and visual congruencies in the displays. It seems that this ability emerges early in development as newborns are already able to match a facial display to the corresponding speech stream (Guellaï et al., 2016).

Another dimension of IDS is found in the body gestures of caregivers, which trigger and enhance speech processing. Indeed, caregivers accompany speech with deictic and iconic gestures when talking about objects and actions to infants (Clark and Estigarribia, 2011; Esteve-Gibert et al., 2016), and highlight referential communication by labeling objects while moving them in synchrony with speech (Gogate et al., 2000; Jesse and Johnson, 2016). The caregivers’ use of co-speech gestures seems to boost infants’ receptive vocabulary and memory skills (Goodwyn et al., 2000; O’Neill et al., 2005; Zammit and Schafer, 2011; Igualada et al., 2017). Igualada et al. (2017) tested preschoolers in a word learning task in which certain words in the list were accompanied by a beat gesture, and results indicated that words co-occurring with gestures were better remembered than gesturally unmarked words.

Yet the impact of Infant-Directed Gestures (or ‘gesturese’) on language development is an unresolved issue. Some studies have found that toddlers learn words better if adults accompany object labels with deictic and symbolic gestures, and direct their gaze toward the object (Booth et al., 2008; McGregor et al., 2009). However, other findings do not support this hypothesis, some results showing an absence or very small effect of parental use of deictic and symbolic gestures on infants’ word learning abilities (Zammit and Schafer, 2011; Puccini and Liszkowski, 2012).

## Multimodal Development of Discourse and Narrative Skills

An interesting aspect of prosody is that it can also convey information about syntax (Nespor and Vogel, 1986, 2007; Langus et al., 2012). For example, one can manipulate prosodic cues to influence how listeners interpret syntactically ambiguous sentences (Lehiste, 1973; Cooper and Paccia-Cooper, 1980; Price et al., 1991; Carlson et al., 2001). These effects emerge very quickly during sentence comprehension (Marslen-Wilson et al., 1992; Warren et al., 1995; Nagel et al., 1996; Kjelgaard and Speer, 1999; Weber et al., 2006). In the visual domain, the so-called beat gestures seem to be also used to process the structure of the speech signal. In languages such as Italian, English, Dutch, or Catalan, beat gestures are temporally aligned with pitch accents and boundary tones (Yasinnik et al., 2004; Krahmer and Swerts, 2007; Esteve-Gibert et al., 2017a; Krivokapic et al., 2017). Guellaï et al. (2014) showed that spontaneous gestures accompanying speech can be perceived as prosodic markers by adults. This evidence goes in the same direction as a model based on Israeli Signed Language (ISL) showing that body positions align with rhythmic manual features of the signing stream to mark prosodic boundaries (Nespor and Sandler, 1999; Sandler, 1999, 2005, 2011, 2012).

Speakers use prosodic means to emphasize new and important information in ongoing discourse, and for signaling the conceptual structure of the utterances in narrations (Swerts and Geluykens, 1994; Gussenhoven, 2004; Baumann and Grice, 2006; Ladd, 2008). Likewise, visual strategies are found to serve similar functions. Articulatory and head gestures enhance the perception of contrastive focus (Dohen and Loevenbruck, 2009; Swerts and Krahmer, 2010; Kim et al., 2014; Prieto et al., 2015), and body gestures such as eyebrow and head movements are produced less often as a marker of the theme than as a rheme marker (Ambrazaitis and House, 2017).

Children develop discourse and narrative skills relatively late. At around 5 years of age, children use adult-like discourse markers, dependent clauses and sentential focus to narrate actions with a coherent structure, and these abilities continue to develop over the next years (Hudson and Shapiro, 1991; Berman and Slobin, 1994; Diessel and Tomasello, 2005; Kallay and Redford, 2016). The question is whether gesture and prosodic markers emerge together with the development of syntactic and lexical markers of conceptual structure. On the gesture side, at ages four to five children use beat gestures to emphasize specific information in the sentence (Nicoladis et al., 1999; Capone and McGregor, 2004; Esteve-Gibert et al., 2017b; Mathew et al., 2017). In narrations, children seem to gesture more when they produce longer sentences with more connectives (Nicoladis et al., 1999; Graziano, 2011, 2014; Colletta et al., 2014), and they use different gesture types depending on the age and the type of discourse they produce (Alamillo et al., 2013). Also, they display better narrative skills in a story retelling game if they have had access to manual beat gestures marking information focus and event boundaries (Vilà-Giménez et al., 2017). On the speech prosody side, children at age five and six are found to use the appropriate pitch accents with the right alignment to signal new information in the discourse (see Chen, 2018 for a review), and in narratives they mark event boundaries through pitch direction and linearity (Kallay and Redford, 2016). While results from the gesture literature seem to suggest that gesture marking of discourse structure is directly correlated with the development of linguistic skills, results are less conclusive from the speech prosody side. Kallay and Redford (2016) propose that the correlation between the development of linguistic skills and the development of discourse structure might occur at the level of local pitch features, while more global aspects of discourse prosody such as slope steepness, pitch resets, or pause duration might be mediated by non-linguistic factors such as breathing.

## Multimodal Cues in Developing Emotion Perception and Production

Perceptual skills related to emotion develop very early in infancy. It has been found that 5-month-old infants are able to distinguish between two different emotions on the basis of the speaker’s facial expressions and the acoustic properties of speech (Fernald, 1993; Grossmann et al., 2006; Vaillant-Molina et al., 2013). Evidence using continuous speech typically shows that young infants rely on the congruence between auditory emotions (happy, angry) and the appropriate facial expressions (Soken and Pick, 1992; Walker-Andrews, 1997). Production-wise, young infants at 4–5 months of age express emotions such as sadness or enjoyment through facial expressions, and at 12 months of age their facial expressions can signal fear, pain, surprise, or interest (Sullivan and Lewis, 2003). At similar ages, vocal cues are also found to reflect their emotional states (Scheiner et al., 2002; Oller et al., 2013; Lindová et al., 2015).

It is not until much later, however, that children use this early sensitivity to visual and acoustic features of emotion to understand their interlocutor’s affective state (Nelson and Russell, 2011; Quam and Swingley, 2012; Berman et al., 2016). Berman et al. (2016) designed a task in which 3- and 5-year-old children had to match pictures of happy-looking and sad-looking faces to happy-sounding and sad-sounding speech, while explicit (pointing) and implicit (eye gaze) responses were measured. Results indicated that only 5 years old children were able to explicitly match the appropriate acoustic and visual cues of emotion, and that at 3 years of age they could only do it implicitly for the negative valence pair.

Even more challenging for children are stimuli in which the speaker intentionally mismatches the audiovisual cues of emotion from the contextual and lexical information, with the purpose of being ironic. In such cases, children at 5–6 years of age tend to interpret the utterance literally even if prosodic cues of emotion signal the speaker’s irony (Nakassis and Snedeker, 2002; Laval and Bert-Erboul, 2005; Aguert et al., 2013; Bosco et al., 2013), and only if the utterance is produced together with visual cues of emotion can children infer non-literal meaning (Gil et al., 2014; González-Fuente, 2017). Taken together, all these findings indicate that vocal and visual cues of emotion are recognized and used very early in infancy, and that children use these early skills to process other people’s emotions once more complex cognitive abilities are in place.

## Acoustic and Visual Markers of Intentions, Attitudes, and Beliefs

Infants recognize and express their social intentions and communicative goals very early in development, and they use prosodic and gestural means to do so. Twelve-month-old infants rely on pitch, duration, and the shape of the gesture (open-palm pointing, index-finger pointing, etc.) to understand whether the interlocutor is communicating in order to request an object, to inform the caregiver about its presence, or to share interest about it (Behne et al., 2012; Sakkalou and Gattis, 2012; Esteve-Gibert et al., 2017c; Rohlfing et al., 2017). For example, 12-month-old infants use the shape of a pointing gesture and the information from the context to understand that their interlocutor is referring to a certain object in space with a specific social intention (Behne et al., 2012). Interestingly, when contextual cues are ambiguous or uninformative, 12-month-old infants use the shape of the pointing gesture in combination with the prosodic features of speech to infer the speakers’ pragmatic intentions (Esteve-Gibert et al., 2017c). Some months later, at around 15 months of age, infants distinguish an action as being accidental or intentional only through the prosodic features of the interlocutor’s speech (Sakkalou and Gattis, 2012).

At these pre-lexical stages of language development, prosody and gesture also enable infants to express their intentions toward their interlocutor. We know that 12-month-old infants produce pointing gestures toward referents in space with the purpose of requesting or declaring information, interest, attitudes, or actions (Tomasello et al., 2007; Kovács et al., 2014). It seems that not only pointing gestures but also the prosodic cues of the vocalizations accompanying them indicate the infants’ intention (Grünloh and Liszkowski, 2015; Aureli et al., 2017). Aureli et al. (2017), for instance, found that when Italian-learning 12- to 18-month-olds intend to produce points with a declarative function, the intonation of the vocalization accompanying these points is mostly falling, while it rises to accompany points aimed at asking objects from the interlocutor (thus paralleling what happens in adult speech).

The speaker’s beliefs and attitudes about the content of the message are also signaled through vocal and visual strategies. Prosodic cues such as speech rate, pitch level and direction, or voice quality, and gestures such as eyebrow furrowing, head tilt, or shoulder shrugging, are reliably markers of the speaker being uncertain, incredulous, or polite (Krahmer and Swerts, 2005; Dijkstra et al., 2006; Crespo Sendra et al., 2013). Children need complex cognitive mental abilities (the so-called ‘Theory of Mind’) to understand and express these meanings in language (Wellman, 1990; Perner, 1991; Gopnik, 1993). A large body of research has dealt with the question of when these abilities emerge. Some researchers propose that only at ages four to five do children have fully developed mind-reading abilities, since it is at this age that they succeed in false-belief tasks (Wimmer and Perner, 1983; Baron-Cohen et al., 1985). Yet others claim that younger infants show early cognitive abilities of this kind when less cognitively demanding tasks are used (Onishi and Baillargeon, 2005; Baillargeon et al., 2010; Kovács et al., 2010). Studies exploring the development of prosodic and gesture cues to interpret the other’s beliefs and attitudes suggest that children’s belief comprehension increases significantly during the preschool years. For example, at 3–5 years of age children detect at above chance level the speaker’s beliefs about what she/he is saying thanks to the speaker’s facial expressions and, interestingly, those that are more accurate are those with more sophisticated belief-reasoning skills (Armstrong et al., 2014). Visual information is found to be a stronger cue for preschoolers than prosodic cues of uncertainty, even if prosody is a stronger indicator still than lexical information (Moore et al., 1993; Hübscher et al., 2017). On the production side, children first use prosody than lexical cues to mark uncertainty in speech (Hübscher et al., 2016), and at 7–8 year of age they signal uncertainty through facial expressions such as eyebrow raising or furrowing or funny faces, and with prosodic cues such as fillers, delays, and high intonation (Krahmer and Swerts, 2005; Visser et al., 2014). All together, these studies suggest that children use the acoustic and visual components of prosody before lexical markers to understand and produce beliefs and attitudes in language. Yet, more studies are required to disentangle which of these prosodic dimensions (visual or acoustic) comes first, and whether this developmental path depends on the child’s cognitive abilities and/or on the specific linguistic meaning that is investigated.

## Discussion

The present review is aimed at highlighting recent discoveries on the developmental integration of speech in the auditory and visual domains, focusing on the prosodic level. Although there are more and more evidence of links between speech and gestures, we do not fully understand the relative weight of each modality in language comprehension, and we need to clarify whether prosody has parallel forms and functions in the acoustic and visual domains. Adopting a developmental approach could help in answering these questions.

Developmental research can help disentangle whether gestures are part of the speakers’ linguistic system. There is consistent evidence that infants and children first use the gesture modality to refer to objects in space before they use words and word-gesture combinations to do so (Bates et al., 1979; Butcher and Goldin-Meadow, 2000; Esteve-Gibert and Prieto, 2014). In fact, the rate of gesturally pointed referents is a reliable sign of the infants’ vocabulary skills at later stages (Rowe and Goldin-Meadow, 2009; Igualada et al., 2015), and the rate of pointing-speech combinations at 18 months of age (when pointing and speech provide complementary meanings) is a reliable predictor of sentence complexity at 42 months of age (Rowe and Goldin-Meadow, 2009). Mathew et al. (2017) observed that 6-year-olds produce ‘beat’ gestures with an emphasizing function, but surprisingly the gesture-accompanying words did not always bear a pitch accent, suggesting that children are still learning to use the speech modality to emphasize discourse elements, while they seem to already master the gesture. Although not all language functions emerge first in the visual modality (note, for instance, that toddlers first express actions with verbs and only later are able to represent that same action with iconic gestures depicting that action; Özçaliskan et al., 2003), the abovementioned results indicate that infants and children do use gestures for linguistic purposes, and that speech and gestures might be part of the same linguistic and communicative system (Kendon, 1980; McNeill, 1992; Goldin-Meadow, 1998).

It is still an open question the reason why certain linguistic functions are first expressed through gestures and some others are first observed in the acoustic dimension. Parladé and Iverson (2011) propose a dynamic systems approach to cope with the fact that infants prefer to use one modality over the other for a given linguistic function at certain stages in language development. According to these authors, in periods where infants increase their skills in one communicative behavior, there might be a temporary regression in an alternative communicative behavior. For instance, the authors find that when infants’ vocabulary increases, their production of multimodal communicative behaviors (i.e., combination of vocal, gestural, and affect behaviors) is reduced. Later, once vocabulary skills are stabilized, the rate of multimodal communicative behaviors increases again. It remains unclear, however, why certain linguistic functions emerge first through gesture rather than through speech, and vice-versa, as well as what motor, cognitive, or communicational factors might influence this behavior.

Studies in brain imagery could also help tease apart the possibility of a gesture/speech linkage in language. Indeed, in adult populations, it has been shown that listening to speech evokes neural responses in the motor cortex. This has been controversially interpreted as evidence that speech sounds are processed as articulatory movements (Pulvermüller and Fadiga, 2010). Recently, Biau et al. (2016) evaluated beat synchrony against arbitrary visual cues bearing equivalent rhythmic and spatial properties as the gestures. Their results revealed that left Middle Temporal Gyrus and Inferior Frontal Gyrus were specifically sensitive to speech synchronized with beats, compared to the arbitrary vision–speech pairing. Hence, it seems that co-speech gestures and speech perception are instantiated through a specialized brain network sensitive to the communicative intent conveyed by the speaker’s whole body.

There are very few studies investigating the developmental signs of the vocal-motor linkages at the neural level, and most evidence comes from populations with developmental disorders and brain injuries. For instance, children with perinatal brain lesions are found to have both lower rates of gesture production and smaller vocabularies (Sauer et al., 2010). Another way to specify the links between gestures and speech would be to explore how sensorimotor feedback influences auditory-visual speech processing, for instance by investigating whether the production of gestures influences infants’ speech fluency. If more evidence is obtained showing that gesture and speech mutually influence each other in language production, perception, and comprehension, this would suggest that they are part of the linguistic system and not only communicative means, especially in development.

Among the linguistic aspects revealing the gesture/speech link more clearly, we have shown that prosody has a prominent status. Prosodic targets are anchoring points for manual gestures and facial expressions to align, pitch accents attracting prominent gestural phases and prosodic phrase boundaries framing the scope of gesture movements. This is true in adults (Hadar et al., 1983; De Ruiter, 1998; Leonard and Cummins, 2011; Esteve-Gibert and Prieto, 2013; Ferré, 2014; Ishi et al., 2014; Ambrazaitis and House, 2017; Esteve-Gibert et al., 2017a), and it also seems to hold for infants and children (Butcher and Goldin-Meadow, 2000; Esteve-Gibert and Prieto, 2014; Mathew et al., 2017). While more research is needed to examine the patterns of this temporal linkage in infants’ productions (especially in stages when these prosodic targets become adult-like), perception studies show that infants are sensitive to the alignment of prosodic and visual cues as early as 8–9 months of age (Kitamura et al., 2014; Esteve-Gibert et al., 2015). It has been proposed that the driving force of this temporal linkage is a bi-directional influence between gesture and speech ‘pulses’ (i.e., peaks in an ongoing rhythm) (McNeill, 1992; Tuite, 1993; Iverson and Thelen, 1999; Port, 2003; Rusiewicz and Esteve-Gibert, 2018).

Prosody and gestures also overlap in terms of which linguistic functions they are used for. Infants use visual correlates of prosody to segment the speech stream (e.g., Kitamura et al., 2014; Guellaï et al., 2016), to organize information at the discourse level (e.g., Nicoladis et al., 1999; Capone and McGregor, 2004; Mathew et al., 2017), and to express emotions, intentions, and beliefs (Sullivan and Lewis, 2003; Esteve-Gibert and Prieto, 2014; Berman et al., 2016; Aureli et al., 2017; González-Fuente, 2017). Children are sensitive to the fact that visual cues convey relevant linguistic meaning, and experimental evidence shows that gestures are processed earlier and more accurately than prosodic or lexical cues (Armstrong et al., 2014; Esteve-Gibert et al., 2017c; Hübscher et al., 2017). If future studies confirm that infants and children first process through visual cues what they later learn to process acoustically, this would mean that gestures are key in the development of linguistic categories, and that they not only precede but also scaffold language development (see a proposal on this regard in Hübscher et al., 2017). Furthermore, by examining in more detail how visual and acoustic cues of prosody emerge, evolve, and interact across development, we will be able to develop models that can predict and guide intervention in the case of atypical language development. The studies reviewed here have shown that gestures are tightly linked to prosody at the formal and functional levels and across different stages of language development. Still, further studies are needed to fully clarify the origin of these links and their implications for language acquisition.

## Author Contributions

All authors have equally participated to the discussion and writing of the manuscript. BG has had a leading role in section 1 (Introduction), section 3 (Word Learning), and section 7 (Discussion), while NE-G has had a leading role in section 2 (Temporal Aspects), section 4 (Narrative Skills), section 5 (Emotions), and section 6 (Intentions, Attitudes, and Beliefs).

## Conflict of Interest Statement

The authors declare that the research was conducted in the absence of any commercial or financial relationships that could be construed as a potential conflict of interest.
